# Aerosol Inhalation Delivery of Ag Nanoparticles in Mice: Pharmacokinetics and Antibacterial Action

**DOI:** 10.3390/antibiotics12101534

**Published:** 2023-10-12

**Authors:** Sergey V. Valiulin, Andrei A. Onischuk, Alexandra P. Pyryaeva, Sergey V. An’kov, Anatoly M. Baklanov, Nikolay N. Shkil, Ekaterina V. Nefedova, Kirill S. Ershov, Tatyana G. Tolstikova, Galina G. Dultseva

**Affiliations:** 1 Voevodsky Institute of Chemical Kinetics and Combustion SB RAS, 3 Institutskaya Str., 630090 Novosibirsk, Russia; 2 Vorozhtsov Institute of Organic Chemistry SB RAS, 9 Lavrentyev Ave., 630090 Novosibirsk, Russia; 3 Siberian Federal Scientific Center of Agro-BioTechnologies RAS, 630501 Krasnoobsk, Russia

**Keywords:** aerosol inhalation delivery, silver nanoparticles, pharmacokinetics, antibacterial activity

## Abstract

The aerosol inhalation delivery of composite particles consisting of Ag nanoparticles enveloped by polyvinylpyrrolidone was investigated in experiments with mice. An ultrasonic nebulizing system was created for the generation of aerosols with a mean diameter and mass concentration of 700 ± 50 nm and 65 ± 5 mg/m^3^, respectively. The mass fraction of Ag in the composite particles was *α* = 0.061. The aerosol delivery was performed in a whole-body chamber with an exposition time of 20 min. Pharmacokinetic measurements were taken and the silver concentrations in the blood and lungs of the mice were measured as a function of time after exposition by means of electrothermal (graphite furnace) atomic absorption spectrometry. The inhalation dose and other pharmacokinetic parameters were determined. The antibacterial effect of aerosolized silver was assessed for mice infected with *Klebsiella pneumoniae 82* and *Staphylococcus aureus ATCC 25953*. The survival rate of the infected mice after the aerosol exposure demonstrated the high antibacterial efficiency of Ag nanoparticles after inhalation delivery.

## 1. Introduction

Silver has been applied as a remedy since ancient times. Silver vessels were used to preserve water and food as early as 4000 BC. Silver preparations were used by the ancient Greeks to treat ulcers, and silver plates were applied to achieve better wound healing. In the Middle Ages, medical applications included silver wire or coated suture. Colloidal silver and silver nitrate were used for better wound and ulcer healing, for dental hygiene, to prevent ophthalmia neonatorium in newborn infants, to treat surgical infections, and for other applications. In the first half of the twentieth century, the intravenous administration of colloidal silver was commonly used [1,2,3,4,5]. The discovery of antibiotics in the 1940s interrupted the use of silver against microbial infections. However, the emergence of antibiotic-resistant bacteria within recent decades has revived interest in the antibacterial and antiviral properties of silver [6,7,8,9,10]. 

Although the antibacterial and antiviral activity of silver is well known, the exact mechanism underlying this activity is still unresolved. There are three mechanisms proposed in the literature through which silver nanoparticles act on bacteria. One of them is Ag particle bonding to the cell membrane. Another accepted mechanism is particle adherence to bacterial cell walls and subsequent particle absorption into the cell causing damage to the intracellular structures. The third mechanism includes the interaction of Ag particles with bacteria, resulting in the generation of reactive oxygen species, which causes cellular damage [11,12,13,14,15,16]. In addition, Ag^+^ ions contribute significantly to cytotoxic activity. Ag^+^ ions interact with respiratory chain proteins on the cell membrane and interrupt the reduction of molecular oxygen in cells, thus inducing cellular oxidative stress through the production of reactive oxygen intermediates [17].

The toxic effects of silver particles in humans, including possible damage caused by silver absorption in tissues, have not yet been well studied. It is suggested that Ag^+^ ions released from the surface of particles bind with chloride and phosphate anions or form complexes with albumins or macroglobulins. These bound ions can result in a toxic effect [5,18]. However, it is accepted that health risks associated with the systemic absorption of silver are low enough [5,18,19].

Respiratory tract infections are among the most common causes of human illness [20]. Effective antimicrobial therapy requires high drug concentrations at the target site. The inhalation delivery of antibacterial agents allows a higher concentration to be deposited directly in the lungs than when delivered systemically, thus minimizing the systemic toxicity [21,22]. Therefore, pulmonary drug delivery has become an attractive target in the health care research area [22,23,24,25]. Due to their unique antiviral and antibacterial properties, silver nanoparticles are considered promising candidates for respiratory tract infections. However, the toxicity of inhaled silver nanoparticles is a matter of great concern. In this regard, there are many publications studying the possible adverse effects from the inhalation of silver nanoparticles [25,26,27,28,29,30,31,32,33,34,35,36]. As demonstrated in the comparative studies of biological effects caused by different silver forms (vapor, nanoparticles, etc.), silver nanoparticles exhibited substantially lower toxicity than silver ions or fine dusts [37]. It was found that the prolonged inhalation of silver nanoparticles did not result in considerable negative health effects. One of the promising applications of antibacterial inhalation therapy is in the treatment of tuberculosis (TB), which continues to be a major health concern. It was found that silver nanoparticle inhalation exhibited good efficiency against TB mycobacteria [6,7,8]. However, the biological activity of aerosolized silver was determined to depend on the silver particle size, which makes this parameter necessary in assessing the curative effects of silver nanoparticles [38].

The objective of this paper is to elaborate an experimental approach for silver aerosol delivery in laboratory mice, which includes supersonic nebulization, mice exposure in a whole-body (WB) inhalation chamber with a controlled inhalation dose, the measurement of pharmacokinetic parameters, and the evaluation of silver particles’ antibacterial efficiency in experiments with outbred male mice infected with the archival strain of *Klebsiella pneumoniae* 82 and *Staphylococcus aureus ATCC 25953*.

## 2. Experimental

### 2.1. Aerosol Generation and Inhalation Equipment

**Figure 1:**
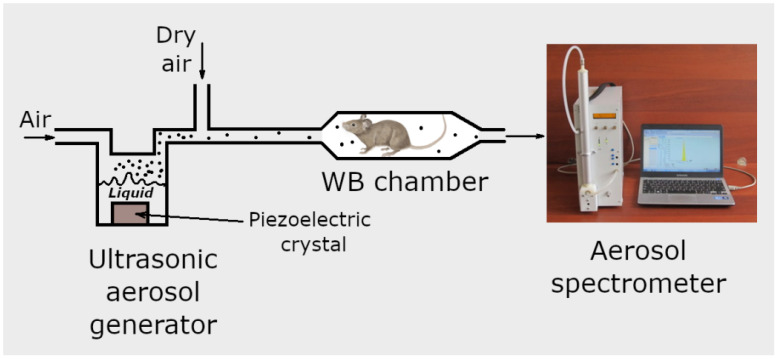
Schematic of inhalation system.

To generate aerosol, the food supplement “Argovit-C” is used, which is a suspension of silver nanoparticles stabilized with polyvinylpyrrolidone (PVP) in water, with a concentration of 2.0 mg/mL. For this purpose, 5 mL of colloidal solution is poured into the ultrasonic nebulizer chamber equipped with a piezoelectric crystal (Figure 1). The crystal vibrates at a frequency of 1.7 MHz, so that the formed sound waves promote the generation of droplets of the aqueous solution of silver stabilized with PVP. Filtered air passes through the nebulizer chamber at a flow rate of 0.2 L/min, creating the outlet aerosol flow. The outlet aerosol flow is then diluted with dry pure air. The flow rate of the diluting air is 2.8 L/min, and its humidity is less than 2.5%. As water vapor is undersaturated in the final aerosol flow, water evaporates from drops within a few milliseconds, and the resulting dry silver/PVP particles are supplied to the WB inhalation chamber in which laboratory mice are placed. The experimental procedure is described in more detail by Valiulin et al. [39,40]. 

The WB exposition chamber, made of a quartz cylinder, is 2.0 L in volume, 31.0 cm long, and equipped with stainless steel ports at both ends. In the exposition chamber, the animals are immersed in the aerosol atmosphere and are free to move. During the exposition, 6 to 10 mice are housed in the WB chamber, and the rate of aerosol supply to the chamber is 3.0 L/min. 

The aerosol particle size and number concentration in the inhalation chamber are monitored with the aerosol spectrometer [41,42,43] designed and built at the Voevodsky Institute of Chemical Kinetics and Combustion, Novosibirsk, Russia. The aerosol spectrometer consists of a diffusion battery, condensation chamber, and photoelectric counter. The ranges of particle diameter and number concentration are 3.0–1100 nm and 10^1^–5·10^5^ cm^−3^, respectively, in direct measurements (without aerosol diluter). Using the aerosol diluter, the aerosol concentration can be measured up to 10^9^ cm^−3^ [44]. The particle size distribution and mean particle diameter, measured with the help of the diffusion battery, are compared with the values determined with a JEM 1400 transmission electron microscope (TEM). Sampling for TEM is performed using special grids for electron microscopy, covered with a polyvinyl formal film, using a one-cascade vacuum impactor or thermoprecipitator [45].

Pharmacokinetic experiments were carried out with outbred laboratory male mice CD-1 (body weight 23 ± 2 g). These mice were obtained from the SPF vivarium of the Federal Research Center Institute of Cytology and Genetics of the Siberian Branch of the Russian Academy of Sciences. The animals were housed in cages at 22–25 °C on a 12 h light–dark cycle and had free access to food and water. The lungs and blood of the laboratory animals were collected immediately after the mice were sacrificed through cervical dislocation. Then, the lungs were homogenized with an ultrasonic tissue grinder (QSonica Sonicators Q55). The mass of silver in the blood and lungs was determined by means of electrothermal (graphite furnace) atomic absorption spectrometry with a Solaar M6 Atomic Absorption Spectrometer instrument. 

The total dose *D* of silver (per kilogram of body weight) delivered in the inhalation experiments is determined as

(1) $$D(mg/kg)=\frac{1}{m}C_{A}\varepsilon v_{m}t_{inh}\alpha$$

where *m* is the mean mass (in kg) of mice involved in the inhalation experiment, *C_A_* (mg/cm^3^) is the aerosol mass concentration in the exposition chamber, *ε* is the total particle deposition efficiency in the respiratory tract, determined as the number of particles deposited in the respiratory airways divided by the number of particles that were present in this air volume in the exposition chamber, *v_m_* (cm^3^/min) is the minute volume, i.e., the total volume inhaled by a mouse per minute, *t_inh_* (min) is the inhalation time, and *α* is the mass fraction of silver in aerosol particles. The quantity *v_m_* can be estimated for the mice in WB chambers as [46]

(2) $$v_{m}({cm}^{3}/min)\approx \left(800\pm 100\right){\left(m/kg\right)}^{0.75}$$

The pulmonary deposition efficiency *ε* can be expressed as a sum of two Gaussian functions [46]

(3) $$\varepsilon (\ln(d_{m}))=0.85\exp\left(-\frac{1}{2}{\left(\frac{\ln\left(d_{m}/4.0(nm)\right)}{2.2}\right)}^{2}\right)+0.60\exp\left(-\frac{1}{2}{\left(\frac{\ln\left(d_{m}/1590(nm)\right)}{1.1}\right)}^{2}\right)$$

where *d_m_* is the arithmetic mean diameter of particles.

### 2.2. Investigation of Antibacterial Activity

The antibacterial effect of the silver nanoparticles after their pulmonary administration was studied with the murine bacterial sepsis model in outbred CD-1 mice. Female mice at the age of 60 days, with body weight 25 ± 2 g, were used in the experiments. All manipulations with the animals were carried out according to the Order of the Ministry of Health of the RF No. 199n of 1 April 2016 “Guidelines of Good Laboratory Practices” and the provisions of Directive 2010/63/EU of the European Parliament and Council of the European Union of 22 September 2010 on the protection of animals used for scientific purposes.

Bacterial sepsis was provoked in the mice through the intraperitoneal injection of the bacterial suspension with a concentration of 10^6^ CFU/mL in the amount of 0.5 mL per mouse. The studies involved two bacterial strains: *Klebsiella pneumoniae* 82 (Gram-negative) and *Staphylococcus aureus ATCC 25953* (Gram-positive).

The experiment with *Klebsiella pneumoniae* 82 bacteria included 60 animals divided into three groups, with 20 animals in each group. The number of animals involved in the experiment with *Staphylococcus aureus ATCC 25953* bacteria was 30, divided in a random manner into three groups; each group included 10 animals. The animal groups and related manipulations are described in Table 1. The animals of all groups, 10 mice per cage, were kept under standard conditions with free access to water and food under a 12 h day–night cycle. The scheme of inhalation treatment is shown in Table 2. Inhalation was carried out within one day.

The efficiency of the treatment with inhaled silver nanoparticles was estimated relying on the survival rate over 9 days and on the results of murine blood inoculation on nutritional medium. For inoculation, several animals from each group were withdrawn from the experiment through cervical dislocation an hour after the last inhalation. Blood was sampled in the amount of 0.1 mL from the open heart. Each blood sample was placed in 1 mL of 0.85% aqueous NaCl solution; 0.1 mL of the obtained solution was uniformly applied on meat infusion agar in a Petri dish 90 mm in diameter. Incubation was carried out for 24 h at a temperature of 37.5 ± 0.5 °C. The grown colonies were counted manually.

## 3. Results and Discussion 

### 3.1. Aerosol Measurements

**Figure 2:**
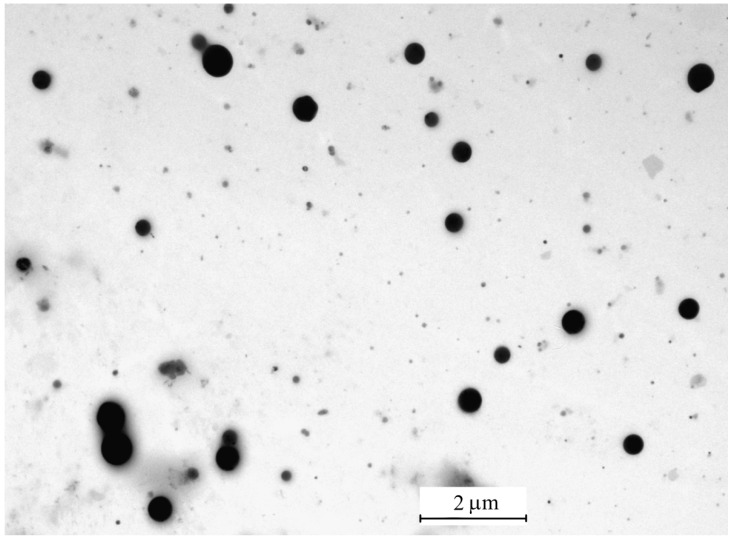
TEM image of dried aerosol particles formed by ultrasonic nebulizer.

**Figure 3:**
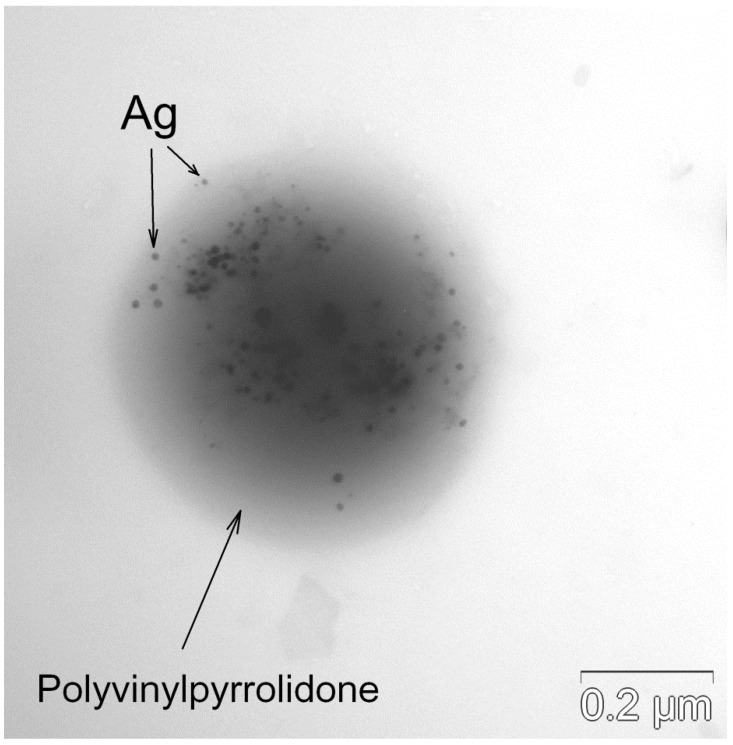
Single composite particle consisting of Ag nanoparticles enveloped with polyvinylpyrrolidone.

**Figure 4:**
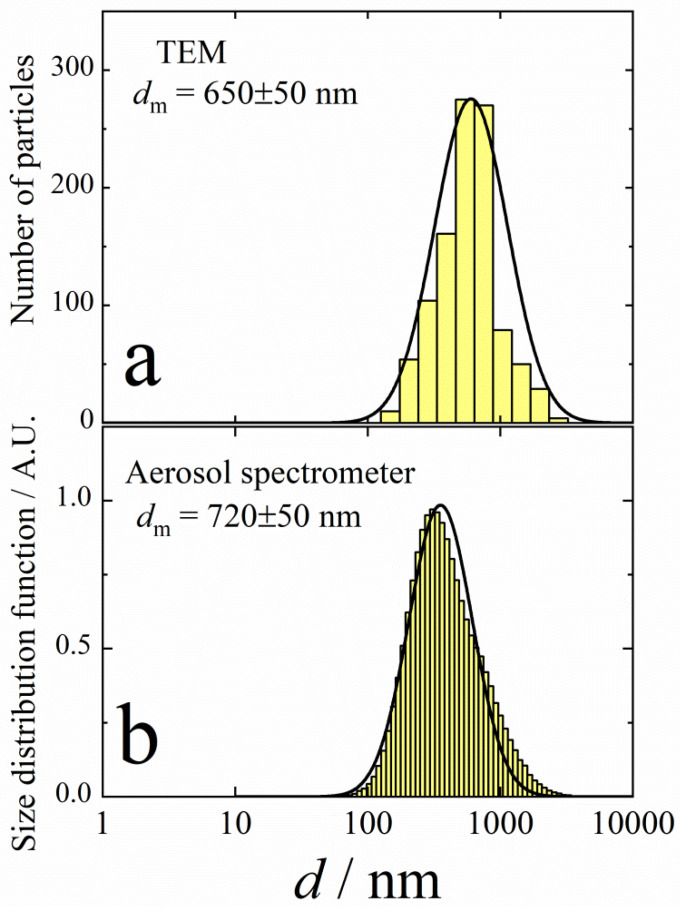
Frequency distribution over diameters of composite particles formed by ultrasonic aerosol generator. (**a**)—TEM analysis; (**b**)—aerosol spectrometer. Solid lines are lognormal functions with the standard geometric deviation *σ*_g_ = 1.7.

The inhalation delivery is provided using ultrasonic aerosol generator, which converts electrical energy to high-frequency vibrations. The vibrations are transferred to the surface of the liquid solution, creating standing waves. These waves break the liquid into small drops, which are carried with the air flow that passes through the generator. The initial composition of drops is the same as that of original solution. On the way out of the ultrasonic generator, the air flow with drops is mixed with the diluting flow of dry air. In the dry medium, the drops evaporate within several milliseconds [37]. A TEM image of the final aerosol particles is shown in Figure 2. These particles consist of Ag nanoparticles enveloped with polyvinylpyrrolidone. An example of such a composite particle is given in Figure 3. The frequency distributions over particle diameter *d*, as determined from the aerosol spectrometer and TEM images, are shown in Figure 4. The arithmetic mean diameter of the composite particles is 700 ± 50 nm. The size distributions are fairly approximated using the lognormal function with the standard geometric deviation *σ*_g_ = 1.7:

(4) $$f(d)=\frac{1}{\sqrt{2\pi }d\ln\sigma_{g}}\exp\left(-\frac{1}{2}\frac{{\left(\ln\frac{d}{d_{0}}\right)}^{2}}{{\left(\ln\sigma_{g}\right)}^{2}}\right)$$

where *d*_0_ is the mean geometric diameter.

The mass fraction of silver in composite particles is determined by sampling the particles using the Petrianov high-efficiency aerosol filter [47]. The total particle mass is obtained through weighing, and the mass of silver is determined by means of electrothermal (graphite furnace) atomic absorption spectrometry. The silver mass fraction was determined *α* = 0.061. Taking into account that the densities of silver (*ρ_Ag_*) and polyvinylpyrrolidone (*ρ_Poly_*) are 10.49 and 1.19 g/cm^3^, respectively, the equivalent density (*ρ_eq_*) of composite particles can be evaluated as

(5) $$\rho_{eq}={\left(\frac{\alpha }{\rho_{Ag}}+\frac{\left(1-\alpha \right)}{\rho_{Poly}}\right)}^{-1}=1.26$$

This value of *ρ_eq_* is used to determine the inhaled aerosol mass concentration from the size distribution and number concentration, determined with the help of the aerosol spectrometer.

**Figure 5:**
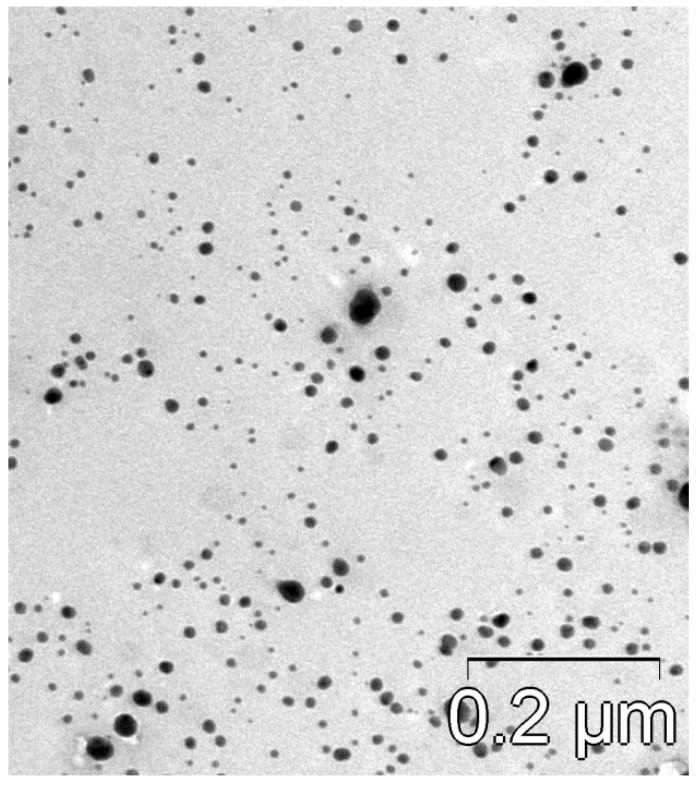
TEM image of Ag nanoparticles.

**Figure 6:**
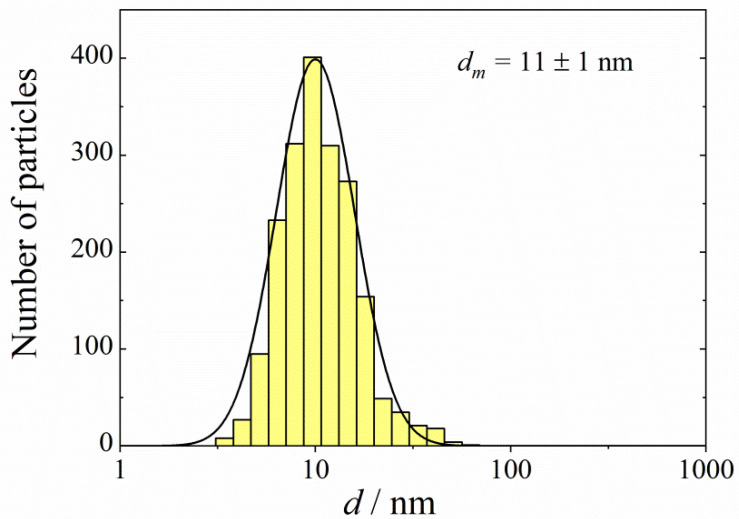
Frequency distribution over diameters of silver nanoparticles. Solid line follows the lognormal function with the standard geometric deviation *σ*_g_ = 1.5.

A TEM image of Ag nanoparticles is shown in Figure 5. The size distribution of the silver particles, as determined from the elaboration of the TEM images, is shown in Figure 6. The size spectrum is well approximated using the lognormal function with *σ*_g_ = 1.5. The arithmetic mean diameter of the silver nanoparticles is dm = 11 ± 1 nm.

### 3.2. Pharmacokinetics of Silver Particles

**Figure 7:**
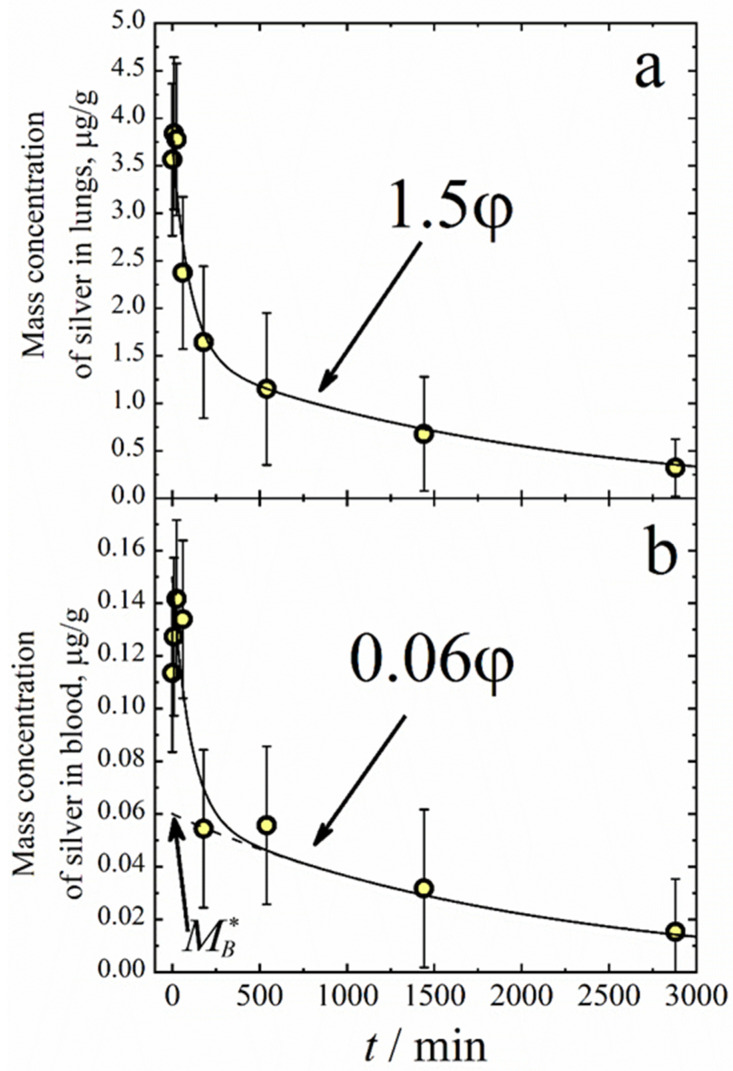
Mass fractions of silver in lungs (**a**) and blood (**b**) vs. time after inhalation. Solid lines follow the double exponential decay function $\varphi =1.5*exp(-k_{1}t)+exp(-k_{2}t)$.

The pharmacokinetic measurements involved 30 animals. The aerosol inhalation was performed in the WB chamber for 20 min. The aerosol mass concentration was 65 ± 5 mg/m^3^ during the exposition, and the arithmetic mean diameter was *d_m_* = 700 ± 50 nm. The mass of silver delivered through inhalation during the exposition was 1.9 μg per mouse, which equaled the dose per kg of body weight *D* = 85 ± 10 μg/kg. Each animal was used only once in the inhalation procedure. Then, the animals were extracted from the chamber and killed via cervical dislocation after some delay, time *t*. The blood and lung samples were collected immediately after the mice were sacrificed. Figure 7 shows the silver concentration in the lungs and blood as a function of time *t* after aerosol exposition. One can see that immediately after the exposition, the mass concentration of silver in the lungs is 3.7 μg/g. The mean mass of the lungs was measured as 0.245 ± 0.040 g, and the mean mass of the silver deposited in the lungs was 0.245 × 3.7 = 0.91 ± 0.10 μg. On the other hand, the total dose of silver inhaled was determined as 1.9 μg per mouse, and the lung-deposited fraction for the particle diameter 700 nm was about 45% of the total number of particles deposited in the respiratory airways (extrathoracic region and lungs) [48]. Therefore, from the total inhalation dose, one should expect the mass of silver in the lungs to be 1.9 × 0.45 = 0.86 ± 0.10 μg. In other words, the mass of silver in the lungs is in good agreement with the inhalation dose. The temporal dependence of both the mass of silver in the lungs and that in the blood can be approximated using the double exponential decay function.

(6) $$\varphi =1.5*exp(-k_{1}t)+exp(-k_{2}t)$$

where *k*_1_ = 10^−2^ min^−1^ and *k*_2_ = 5 × 10^−4^ min^−1^ are effective first-order rate constants for the initial and final stages of silver elimination. The fact that both masses of silver in the lungs and blood follow the same function *φ* means that the equilibrium between the silver in the lungs and blood occurs very quickly. Taking into account the fact that the total mass of blood is about 1300 mg, and that of the lungs is 245 mg, it is easy to estimate from Figure 7 the equilibrium constant *K_L_* = M_L_/M_B_ = 4.7 ± 1.0, where M_L_ and M_B_ are the mass of silver in the lungs and blood, respectively. 

**Figure 8:**
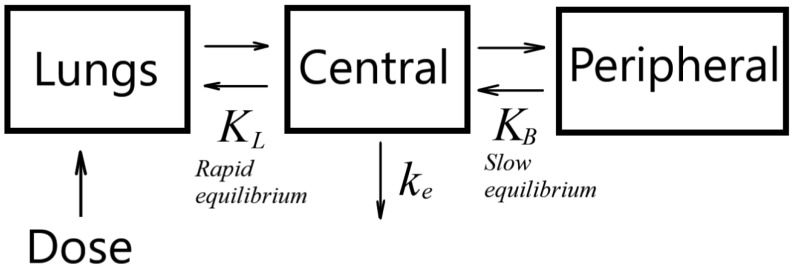
Schematic of three-compartment model.

Silver elimination kinetics can be described with a three-compartment model (Figure 8). In this model, a body is assumed to consist of three compartments: lungs, the central compartment formed by blood and well-perfused organs (heart, liver, spleen, kidneys), and a peripheral compartment consisting of muscles, skin, fat, and other tissues. A typical feature of the three-compartment model is an initial rapid equilibrium between the lungs and the central compartment, and a long-lasting phase of silver distribution over the peripheral compartment. Therefore, the silver elimination kinetics can be divided into two phases: initial (up to about 100 min), when silver is not distributed yet over the peripheral compartment, but there is equilibrium between the lungs and central compartment; and the final stage, when there is total equilibrium. The kinetic equation for the initial stage is

(7) $$\frac{dM_{B}}{dt}=\frac{k_{e}}{K_{L}+1}M_{B}$$

where *k_e_* is the elimination rate constant. The solution of Equation (7) is

(8) $$M_{B}=M_{B}^{0}exp(-\frac{k_{e}}{K_{L}+1}t)$$

where $M_{B}^{0}$ is the mass of silver in the blood just after the end of exposition. Equation (8) determines the initial decay of silver in the lungs. On the other hand, this initial decay follows the first exponential term in Equation (6). Therefore, we have

(9) $$\frac{k_{e}}{K_{L}+1}=k_{1}$$

Taking into account that *K_L_* = 4.7 and *k*_1_ = 10^−2^ min^−1^, we obtain the elimination rate constant from Equation (8) *k_e_* = 5.7 × 10^−2^ min^−1^. The kinetics equation for the final stage of total equilibrium is

(10) $$\frac{dM_{B}}{dt}=\frac{k_{e}}{K_{L}+K_{B}+1}M_{B}$$

where $K_{B}=\frac{M_{P}}{M_{B}}$ is the equilibrium constant for the slow equilibrium between the central and peripheral compartments, and *M_P_* is the mass of silver in the peripheral compartment. The solution of Equation (10) is

(11) $$M_{B}=M_{B}^{*}exp(-\frac{k_{e}}{K_{L}+K_{B}+1}t)$$

where $M_{B}^{*}$ is an effective initial mass of silver in the blood (see Figure 7). Equation (11) determines the final decay of silver in the blood when the total equilibrium occurs. As the final decay follows the second term in Equation (6), we obtain

(12) $$\frac{k_{e}}{K_{L}+K_{B}+1}=k_{2}$$

or

(13) $$K_{B}=\frac{k_{e}}{k_{2}}-(K_{L}+1)\approx 110$$

The distribution volume *V_d_*, i.e., the theoretical volume that would be necessary to contain the total amount of silver at the same concentration as that observed in the blood, can be determined as

(14) $$V_{d}=V_{B}(1+\frac{M_{L}}{M_{B}}+\frac{M_{P}}{M_{B}})=V_{B}(1+K_{L}+K_{B})=V_{B}\frac{k_{e}}{k_{2}}=1.3\frac{5.7\times 10^{-2}}{5\times 10^{-4}}\approx 150\;\mathrm{mL},$$

where $V_{B}$ ≈ 1.3 mL is the volume of blood in mice.

### 3.3. Antibacterial Activity 

When studying the antibacterial effect of silver nanoparticles, the inhalation procedure was the same as in the pharmacokinetic measurements. The aerosol mean diameter and mass concentration were *d_m_* = 700 ± 50 nm and 65 ± 5 mg/m^3^, respectively, which resulted in the pulmonary-delivered silver dose *D* = 85 μg/kg for the inhalation time of 20 min.

The data on the survival rate for the mice during observation for 9 days are presented in Table 3 and Table 4. One can see in Table 3 and Table 4 that the mortality of non-treated animals (*References «+»* group) was 100% in the case of the *Klebsiella pneumoniae 82* strain, and 83.3% for *Staphylococcus aureus ATCC 25953.* At the same time, the mortality of the mice (*Aerosol* group) treated twice with silver nanoparticle inhalations for 20 min did not exceed 6.7%. 

The inoculations of murine blood (see Table 5 and Table 6) at the end of the experiment demonstrate a substantial reduction in bacterial load: by two orders of magnitude for the animals after inhalation treatment with silver nanoparticles.

The results on the survival rate and blood inoculation provide evidence of the high efficiency of inhalation with silver nanoparticles in the treatment of systemic bacterial infection. In turn, this fact confirms the ability of inhaled silver nanoparticles to penetrate from the lungs into the blood flow and to cause systemic antibacterial action.

## 4. Conclusions

The delivery of silver nanoparticles to outbred mice through inhalation was studied using a specially elaborated inhalation system composed of an ultrasonic aerosol generator, whole-body (WB) exposition chamber, and aerosol spectrometer. The ultrasonic generator produced Ag/polyvinylpyrrolidone aerosol with a mean diameter and mass concentration of 700 ± 50 nm and 65 ± 5 μg/cm^3^, respectively. The aerosol spectrometer measures the aerosol mean diameter and mass concentration and transmits these values as input parameters to the software. As a result, the real-time determination of the pulmonary-delivered dose of silver is provided during the inhalation. The 20 min exposition in the WB chamber resulted in an inhalation dose of *D* = 85 μg/kg. It was found that the temporal dependence of the mass of silver in the lungs and blood after inhalation followed the double exponential decay function, which is explained in terms of the three-compartment model. Based on the pharmacokinetic measurements, it is assumed that there is an initial rapid equilibrium between the lungs and the central compartment (blood and well-perfused organs), and a long phase of silver distribution over the peripheral compartment (skin, muscle, fat, tissues). The antibacterial effect of silver nanoparticle treatment was studied using the murine bacterial sepsis model. Bacterial sepsis was provoked through the intraperitoneal injection of two bacterial strains, *Klebsiella pneumoniae 82* and *Staphylococcus aureus ATCC 25953*. It was found that the mortality of the non-treated animals was 100% in the case of the *Klebsiella pneumoniae 82* strain, and 83.3% for *Staphylococcus aureus ATCC 25953.* However, the mortality of the mice treated with silver nanoparticle inhalations did not exceed 6.7%. Thus, the aerosol delivery of silver nanoparticles demonstrated high efficiency in the therapy of systemic bacterial infection.

## Figures and Tables

**Table 1:** Description of the experimental groups of animals.

Group	Actions
**Reference «−»**	Not infected. Inhalation with pure air, 2 times for 20 min.
**Reference «+»**	Infected. Inhalation with pure air, 2 times for 20 min.
**Aerosol**	Infected. Inhalation with silver/PVP aerosol, 2 times for 20 min.

**Table 2:** Inhalation treatment procedure.

Time	Manipulations
0 h 00 min	Introduction of the bacterial suspension (0.5 mL, 10^6^ CFU/mL)
0 h 10 min	Aerosol inhalation for 20 min
3 h 30 min	Aerosol inhalation for 20 min
4 h 30 min	Several animals are withdrawn from the experiment

**Table 3:** Survival rate parameters for animal groups (*Klebsiella pneumoniae 82*).

	Number of Animals	Day 1		Day 2		Day 3		Day 4		Days 5–9	
		A	D	A	D	A	D	A	D	A	D
**Reference «−»**	15	15	0	15	0	15	0	15	0	15	0
**Reference «+»**	15	15	0	7	8	0	15	0	15	0	15
**Aerosol**	15	15	0	14	1	14	1	14	1	14	1

A—alive, D—dead.

**Table 4:** Survival rate parameters for animal groups (*Staphylococcus aureus ATCC 25953)*.

	Number of Animals	Day 1		Day 2		Day 3		Day 4		Days 5–9	
		A	D	A	D	A	D	A	D	A	D
**Reference «−»**	6	6	0	6	0	6	0	6	0	6	0
**Reference «+»**	6	6	0	3	3	2	4	1	5	1	5
**Aerosol**	6	6	0	6	0	6	0	6	0	6	0

A—alive, D—dead.

**Table 5:** Parameters of murine blood inoculation (*Klebsiella pneumoniae* 82).

Animal No.	CFU/mL		
	Reference «−»	Reference «+»	Aerosol
1	0	14,800	200
2	0	12,800	0
3	0	9000	400
4	0	14,500	100
5	0	4200	0

**Table 6:** Parameters of murine blood inoculation (*Staphylococcus aureus ATCC 25953*).

Animal No.	CFU/mL		
	Reference «−»	Reference «+»	Aerosol
1	0	38,500	0
2	0	11,700	0
3	0	36,000	100
4	0	9600	100

## Data Availability

The data presented in this study are available on request from the corresponding author. The data are presented herein in full.
